# Editorial

**Published:** 1898-01

**Authors:** 


### Editorial.

### SMALLPOX VS. CHICKEN-POX.

"Facts are stubborn things," or rendered otherwise "where ignorance is bliss it is folly to be wise," are the two horns of a dilemma.

It will be remembered that some pertinent interrogatories appeared not long since in one of our daily papers as follows:

"*To Dr. J. F. Alexander:*

"Is the eruption of chicken-pox of a vesicular or pustular nature?

"What are the points of difference in the cutaneous manifestations of chicken-pox and smallpox?

"Will the occurrence of chicken-pox prevent the development of vaccine virus either during the eruption or subsequently?

"Is the period of incubation of the smallpox modified by the early introduction of vaccine virus, so as to arrest the development of pustules?

"Should those who have been exposed to smallpox expect to prevent it by prompt vaccination?

"An early reply to the above will be appreciated by many readers. "J. McF. Gaston, M. D."

It would appear that these questions must have escaped the attention of the party to whom they were addressed were it not that I sent them under cover to him at his office. Another member of the medical profession might have been included with him, had not my consideration for his subordinate position induced me to call upon his chief for an explanation of their joint blunder in heralding their failure to recognize a case of smallpox at a stage when the merest tyro in diagnosis should not have made their mistake in classifying it as chicken-pox. If the questions had been answered correctly, valuable information would have been imparted, by which to direct the investigation of the distinctive symptoms of smallpox in their difference from chicken-pox. But now as this disease has disappeared from our city and its suburban population, I leave each medical man to frame his own answer to the questions. It would have facilitated an understanding of those interrogatories to have added one more and to have asked if chicken-pox ever became so virulent as to communicate confluent smallpox to those who were exposed to it, as occurred in connection with a case so diagnosticated by these two members of the profession. It is not the only time that such errors have resulted in the management of doubtful cases, and the consequences in this instance would have proved more serious had not my timely injunction been carried out by the prompt vaccination of all connected with the school where the case occurred. Some days after the development of the disease in others who had been exposed to the contagion of this patient, I prepared the statement which follows to lay before the public, but circumstances have delayed its appearance until it became more appropriate for the consideration of the medical profession than for the people generally:

"Believing that no private interests can now be compromised and that the welfare of the public will be promoted by a publication of the facts in regard to the case at College Park which was pronounced by me to be smallpox and subsequently declared to be chicken-pox by Drs. J. P. Kennedy and J. F. Alexander, I think it proper to give a correct statement of what has occurred up to date in regard to this case.

"On December 4th, I was called upon by Dr. J. J. Foster to accompany him to College Park to see a young man in the Military School, whose case was diagnosticated by me to be smallpox in the pustular stage. I advised that a test vaccination be made in the case which was done by Dr. Foster on two occasions without any manifestations of the characteristic effects of the vaccine virus, thus verifying that the disease was not chicken-pox, but a genuine case of smallpox.

"In the meantime, Dr. J. P. Kennedy visited the patient, and published that the disease was chicken-pox, and subsequently he and Dr. J. F. Alexander, who had also inspected the patient, issued a card stating that it was the chicken-pox and should not cause alarm.

"I am credibly informed that ten cases have developed since, having the recognized symptoms of varioloid or of smallpox, which can be traced to the patient examined by me on December 4th and diagnosticated then as smallpox.

"My recommendation was to vaccinate at once all those connected with the school, and I have learned that vaccination has been generally carried out with all the residents of the place; so that it is reasonable to infer that only those exposed to contact with the disease before undergoing vaccination will suffer from it. The people may congratulate themselves upon following the advice based upon my diagnosis."

On Dec. 24th, I again visited the building at College Park in which the case had been seen by me on the 4th of December, and found that Dr. Foster had been confined to the building with patients under his care in various grades of smallpox and varioloid, dependent upon the period at which vaccination was resorted to. In one case, vaccination being delayed, confluent smallpox was the result; in four cases there was discrete smallpox; and in six cases varioloid made its appearance after the vaccination ran its regular course in six points of insertion on the night I pronounced the case as smallpox. There were two other boys who left for their homes in other States who have taken smallpox due to their previous exposure to this case, thus making in all twelve cases propagated by the first case.

It may be stated that no case of smallpox or of varioloid has occurred among the population of College Park, outside of the building where this case occurred, and hence there was no other focus from which the disease could be propagated. The development of smallpox in twelve different subjects who had been exposed to this case, proves conclusively that my diagnosis of smallpox was correct.

J. McFADDEN GASTON, M. D.

---

### THE PHYSICAL DEGENERACY OF THE NEGRO.

The physical degeneracy of the negro is the subject of an editorial in a recent number of the *New York Medical Record.*

It contains a quotation from Dr. R. H. Johnson, a colored physician of Savannah, to the effect that the present generation of negroes is distinctly inferior to the preceding and that the cause of the degeneracy lies in carelessness, want of forethought and dissipation characteristic of the race.

As a slave, the negro was valuable property and was carefully looked after; now, being free and equal with the white man, he has shown his inability to stand alone by rapidly degenerating at all points. Slavery to the negro was therefore not an unalloyed curse.

Carelessness, lack of forethought and dissipation are essential characteristics of the blacks and have always been, and are not to be held as immediately precipitating causes of their physical decay.

Venereal diseases and tuberculosis have worked the negro's partial undoing and are still at work.

Negroes mature early and are not virtuous. They early indulge in unbridled sexuality with the inevitable result of sooner or later acquiring gonorrhœa or syphilis. Gonorrhœa sterilizes the woman, syphilis devitalizes the offspring and tuberculosis comes in to close the gloomy scene.

A gentleman of this town, who was for more than twelve years city physician in a ward where negroes were numerous, deliberately asserts that seventy-five per cent. of them are syphilitic, tuberculous, or both.

The greater the admixture of white blood, the smaller the negro's resistance to disease.

The "negro problem" is being slowly solved by busy little bacteria, working a slow but sure decay. Unless the negro becomes the ward of this great and good government, or is transported to some primitive country beyond the pale of civilization and its vices, and allowed to recuperate, his doom is sure and certain. The negro problem will be solved by the forces of nature and not by leather-lunged fanatics who shout themselves hoarse over effete theories of social equality.

---

The recent conviction at Paris of Dr. Laporte for mal-practice in causing death from peritonitis following perforation of the bladder in a maladroit attempt at craniotomy, has set the pace for a similar suit, which, however, proved to be something of a boomerang. Witness the following from the *Progre's Medicale*:

"The court of correction at Montdidier has tried recently a curious suit. In last November Dr. Duquesnel, counselor-general at Roye, received the following letter from a man named Fenillette, formerly a harness-maker of Roye:

"'Sir—In 1887, you confined my wife and in performing craniotomy, by your ignorance and employment of improper instruments, you provoked a peritonitis which caused her death.

"'If you had performed Cæsarian section, you would have saved the child, but as you were in collusion with my mother-in-law to destroy my wife and my child, you preferred craniotomy. In causing the death of my wife and child you have done me an injury for which I ask immediate reparation, and if you do not send me 10,000 francs within forty-eight hours, I will make you answer in a court of law for what you have done. 'FEUILLETTE.'

"The procureur of the republic of Montdidier to whom Dr. Duquesnel handed the letter, instituted an investigation which resulted in the prosecution of Feuillette for attempted blackmail. The trial came to court this week at Amiens. Dr. Duquesnel testified that eight years ago he had been called to attend Feuillette's wife, aged 28; her labor was difficult and failing to effect delivery with forceps, he had recourse to craniotomy. The woman died the next day. The doctor added that he had done nothing for which he could reproach himself. The plaintiff Auguste Feuillette, aged 43, formerly harness-maker of Roye, now the proprietor of a saloon at Amiens, stated to the court, that on account of the trial of Dr. Laporte which resulted in his being sentenced to three months in prison, he believed he had a right to demand of Dr. Duquesnel 10,000 francs damages.

"He maintained to the end that the doctor had conspired with his mother-in-law to cause the death of both mother and child, by which means the mother-in-law would gain possession of the wife's dowry.

"The substitute of the procureur, M. Ducatez, applied to the case Art. 400 of the penal code, and Feuillette was condemned to fifteen days imprisonment."

